# How effective is a SMS reminder service at reducing the rate of patients who do not attend (DNA) GP appointments and can DNA rates be further reduced?

**DOI:** 10.1186/1753-6561-9-S1-A24

**Published:** 2015-01-14

**Authors:** CL Harman

**Affiliations:** 1 Partington Family Practice, Health Centre, Central Rd, Partington, Manchester, UK

## Background

Partington Family Practice is based in one of the most socio-economically deprived areas in Greater Manchester. The practice installed an SMS appointment reminder system in 2009 to decrease the DNA rates of its high proportion of service resistant patients. The aims of this project are to investigate how effective this service has been and consider any future services that could further reduce the practice’s DNA rates.

## Methods

A retrospective audit was conducted on records of patients, who booked appointments with a single full time GP at the practice, to determine the percentage who did not attend appointments before and after the introduction of the SMS reminder system. Average annual DNA rates were compared from 2005 to 2013. DNA data for 2008 - 2009 were compared with data for 2012 - 2013 to see if there were any variations in behaviour of patients of different age groups and gender. The uptake of the SMS reminder system and the quality of the patient records were also analysed to ensure the system was being assessed fairly. A mobile telephone survey of 78 patients from different age categories was conducted to obtain their views on the current SMS reminder service and future services that could be implemented to further reduce DNA rates.

## Results

There was a 3% decline in DNA rates in 2010, which reduced to only 1% in 2013. DNA rates have decreased dramatically for 0 to 15 and 30 to 39 year olds but have increased for 16 to 29 year olds, and 70 and older since the introduction of the SMS reminder system. The practice mobile phone records are 60% accurate for 0 to 15 year olds rising to 96% accuracy for 60 to 69 year olds. Cancelling appointments by SMS was preferred over on-line booking services, with between 90 to 100% interest in those aged 0 to 30. Gender did not have a significant effect on patient DNA behaviour.

## Conclusions

The service is more effective for those aged 40 to 70 than younger patients aged 16 - 29. It would be more effective if it was implemented more widely by introducing a well-advertised ‘opt-out’ service and by maintaining more accurate patient mobile phone records, especially for patients aged 40 or younger. The practice should consider implementing a service to cancel appointments by SMS message, which may further reduce DNA rates, especially for patients aged 16 to 29.

